# Influence of artificial tears on corneal parameter measurement using three different devices: Keratometry and Scheimpflug technology, a randomized trial

**DOI:** 10.1111/aos.17487

**Published:** 2025-03-28

**Authors:** Titus Schug, Thomas Kohnen, Klemens Paul Kaiser, Christoph Lwowski

**Affiliations:** ^1^ Department of Ophthalmology Goethe‐University Frankfurt am Main Germany

**Keywords:** artificial tears, keratometry, tomography, topography

## Abstract

**Purpose:**

To evaluate the effect of artificial tear (AT) application on the repeatability of corneal parameters with three different devices.

**Methods:**

In this prospective, randomized clinical trial, 145 patients were measured with one of three devices in the Department of Ophthalmology, Goethe University, Frankfurt, Germany. Two baseline measurements and four measurements following AT application after 30 s, 2, 5 and 10 min were performed.

**Results:**

Forty‐eight eyes were measured with the IOLMaster 700 (Carl Zeiss Meditec AG, Oberkochen, Germany) and Galilei G6 (Ziemer, Port, Switzerland), and 49 with the Pentacam AXL (Oculus, Wetzlar, Germany) (145 total). Repeatability of the mean corneal power (K_m_) between baseline measurements was high (0.049 ± 0.008 D for IOLMaster, 0.061 ± 0.011 for the Pentacam AXL and 0.065 ± 0.009 for Galilei G6) but significantly variable after 30 s and 2 min for IOLMaster 700 (0.177 ± 0.021 D, *p* < 0.001 and 0.079 ± 0.009, *p* = 0.004) and Galilei G6 (0.163 ± 0.020 D, *p* < 0.001 and 0.119 ± 0.014 D, *p* = 0.003). K_m_ differed significantly from baseline at 30 s, 2 and 5 min with Pentacam AXL (0.215 ± 0.041 D, *p* < 0.001; 0.157 ± 0.025 D, *p* < 0.001 and 0.094 ± 0.012 D, *p* = 0.007). Variability was highest at 30 s and decreased over time.

**Conclusion:**

The use of AT leads to high variability of keratometric measurements for each device. This should be considered after use, and at least 5 min should elapse before measurements.

## 1 INTRODUCTION

Some type of cataract is present in 17.2% of Americans over 40 (‘Prevalence of Cataract and Pseudophakia/Aphakia Among Adults in the United States’ 2004). The most common form of therapy is modern small‐incision cataract surgery, which offers great clinical outcomes with rapid post‐operative recovery and a low complication risk profile (Day et al., 2015). Post‐operative satisfaction is largely dependent on good refractive outcomes, especially in multifocal intraocular lenses (IOLs) (Gibbons et al., 2016; Lee et al., 2005). Brogan et al. showed a post‐operative refraction within 0.5 dioptres (D) for 62% of included eyes (Brogan et al., 2019). However, one factor influencing refractive success is the correct assessment of keratometric values, which, along with the axial length of the eye, are the most important parameters for IOL calculation (Khoramnia et al., 2022; Kieval et al., 2020). Many factors have been shown to influence the measurement of the anterior corneal surface, including corneal pathologies (Gupta et al., 2018; Kreps et al., 2020). In the meantime, however, several studies have shown that changes in the corneal tear film can alter keratometric measurements due to tear film instability with subsequent breakage (de Paiva et al., 2003; Huang et al., 2002; Liu & Pflugfelder, 1999; Rochet et al., 2021). In these studies, the instillation of artificial tears (AT) was shown to reduce changes in keratometric measurements and improve topographic indices (de Paiva et al., 2003; Huang et al., 2002; Liu & Pflugfelder, 1999).

Findings across various studies observing the influence of AT diverge, with some indicating an enhancement in measurement quality, while others suggest no discernible improvement, and yet another subset reporting a decline or worsening in quality (Jensen et al., 2020; Rochet et al., 2021; Röggla et al., 2021).

Currently, there are multiple devices used for pre‐operative assessment of the eye. The most widely applied devices use keratometry or Scheimpflug techniques to measure the cornea. Studies largely focus on the influence of AT on keratometry measurements.

The purpose of this study is to investigate if AT application shortly before the assessment of corneal values has an influence on the accuracy of measurements on three different devices, and if so, whether the different principles used by these devices to measure the corneal surface react differently and for how long.

## 2 MATERIALS AND METHODS

This is a prospective, randomized study conducted at one single centre (Department of Ophthalmology, Goethe‐University, Frankfurt, Germany). Healthy individuals were recruited from November 2020 to February 2022. Proper written informed consent for participation was received from all patients. After recruitment, patients were randomly assigned to one of the three devices, and one eye was randomly selected using a raffle box.

An institutional review board approval (Ethics Committee of Goethe University, Frankfurt, Germany) was obtained, and the study was performed following the tenets of the Declaration of Helsinki.

The study has been registered at ClinicalTrials.gov (ID: NCT06358456).

### 2.1 Inclusion and exclusion criteria

Inclusion criteria were an age of at least 18 years. Exclusion criteria were any prior ocular surgery or trauma, any ocular pathology other than cataract (e.g. Fuchs dystrophy, keratoconus), and contact lens wear within 2 weeks of the study. Patients with a history of dry‐eye disease were excluded.

### 2.2 Artificial tears

The AT used in this study was the ‘ARTELAC EDO’ (BAUSCH + LOMB; Laval, Canada) eyedrops whose active ingredient is hypromellose (1.92 mg/0.6 mL container).

### 2.3 Devices

The used devices were the IOLMaster 700 (Carl Zeiss Meditec AG, Jena, Germany), the Pentacam AXL (Oculus Optikgeräte GmbH, Wetzlar, Germany) and the Galilei G6 device (Ziemer Ophthalmic Systems GmbH, Port, Switzerland).

#### 2.3.1 IOLMaster 700

The IOLMaster 700 uses telecentric keratometry. By projecting light onto the anterior surface of the cornea and analysing the reflection, it can measure the flattest and steepest meridians. Compared to its predecessor, the IOLMaster 500, it is also able to measure the total keratometry by using swept‐source optical coherence tomography (OCT). This way, both the anterior and posterior surfaces are taken into account. In our study, only the telecentric keratometry values were considered.

#### 2.3.2 Pentacam AXL

The Pentacam AXL measures K values through a Scheimpflug camera system by capturing images of the anterior segment. The data are then analysed, and a detailed map of the cornea's curvature is provided. With this approach, both the anterior and posterior surfaces of the cornea are taken into account.

#### 2.3.3 Galilei G6

The Galilei G6 integrates dual Scheimpflug cameras with a Placido disc topography system, providing a comprehensive analysis of the anterior segment of the eye.

#### 2.3.4 Measurements

All devices measure contactless. The measurements were performed following the manufacturer's instructions. All six measurements were performed on the same day. Two before the application of the AT with a 1‐min interruption and four afterwards. These measurements were taken 30 s after the application and after 2, 5 and 10 min. The patient was asked to stand up between the measurements to change position on the device to allow independent measurements.

### 2.4 Outcome measures

After randomization, the patients were divided into three groups based on the three devices: Group I (IOLMaster 700, Carl Zeiss Meditec AG, Jena, Germany), Group G (Galilei G6, Ziemer Ophthalmic Systems GmbH, Port, Switzerland) and Group P (Pentacam AXL, Oculus Optikgeräte GmbH, Wetzlar, Germany). The primary outcome was the comparison between corneal measurements before and after the application of AT with different devices. These measurements consisted of the keratometric (K) value. The K value was compared by calculating the absolute difference in K mean (K_m_) between the baseline measurements and the measurements taken after the AT application.

The secondary outcome was the change in corneal astigmatism.

### 2.5 Statistical analysis

The data were collected and entered manually into an Excel spreadsheet (Version 16.77.1, Microsoft Corporation, Redmond, WA, USA). The double angle plots were created using an Excel spreadsheet provided by Abulafia et al. (2018). Statistical analysis was performed using spss Software (Version 29.0.1.0; IBM Corporation, Armonk, NC, USA). The Kolmogorov–Smirnov test was used to test for normal distribution. The Friedman test was performed to analyse differences in K_m_. If statistical significance was observed, a post‐hoc analysis with either a Wilcoxon signed‐rank test or a paired t‐test was performed. An F‐test was used to test for differences in standard deviation between the two measurements. *p*‐values below 0.05 were considered statistically significant. If needed, the *p*‐values were Bonferroni corrected.

The sample size is based on the calculation by Röggla et al. and was calculated using the G*Power 3.1 Software (Heinrich Heine University Düsseldorf, Germany) (Röggla et al., 2021). A clinically relevant difference of 0.15 D (standard deviation (SD) of 0.4) between the first measurement and one after AT application was assumed, leading to a sample size of 48 eyes per group (power of 0.8 with an *α* of 0.05). One eye per patient was used to avoid statistical bias.

## 3 RESULTS

We included 153 eyes from 153 patients (1 eye per patient). Nine eyes were excluded because of the insufficient quality of the scans. The remaining 145 patients consisted of 48 patients in Group I and Group G, respectively, and 49 patients in Group P. Demographic data are presented in Table 1.

**TABLE 1:** Study population characteristics sorted by devices (*n* = 145).

Parameter	Devices		
	IOLMaster 700	Pentacam AXL	Galilei G6
*N*	48	49	48
Age in years (mean ± SD)	32 ± 13	27 ± 10	29 ± 10
Female sex (%)	50%	51.02%	56.25%
Right eye (%)	50%	51.02%	50%
Axial length in mm (mean ± SD)	23.96 ± 1.45	23.89 ± 1.14	24.01 ± 1.19
K_m_ in D (mean ± SD)	42.59 ± 1.30	43.20 ± 1.36	42.96 ± 1.51
K1 in D (mean ± SD)	42.14 ± 1.35	42.69 ± 1.32	42.49 ± 1.50
K2 in D (mean ± SD)	43.05 ± 1.33	43.72 ± 1.47	43.43 ± 1.57
R_m_ in mm (mean ± SD)	7.80 ± 0.24	7.82 ± 0.24	7.87 ± 0.28
R_1_ in mm (mean ± SD)	7.89 ± 0.25	7.91 ± 0.24	7.95 ± 0.28
R_2_ in mm (mean ± SD)	7.72 ± 0.24	7.73 ± 0.26	7.78 ± 0.28

Abbreviations: D, dioptres; K1, corneal power in the flat meridian; K2, corneal power in the steep meridian; K_m_, mean corneal power; R1, radius of curvature in the flat meridian; R2, radius of curvature in the steep meridian; R_m_, mean radius of curvature.

### 3.1 Baseline measurements

When comparing the corneal values of two baseline measurements against each other, no statistically significant difference was found in any device regarding the radius of curvature in the steep meridian (R1) and the flat meridian (R2), Corneal curvature (K), astigmatism and corneal thickness. Detailed statistics are shown in Table 2.

**TABLE 2:** Difference in corneal values between the two baseline measurements without intervention sorted by device (IOLMaster 700, Pentacam AXL, Galilei G6) (*n* = 145).

	IOLMaster 700 (*n* = 48)	Pentacam AXL (*n* = 49)	Galilei G6 (*n* = 48)
R1 in mm (mean ± SD)	0 ± 0.02	−0.002 ± 0.017	0.003 ± 0.02
R2 in mm (mean ± SD)	0.002 ± 0.02	0.012 ± 0.097	0.007 ± 0.02
K1 in D (mean ± SD)	0.001 ± 0.1	0.012 ± 0.097	−0.013 ± 0.109
K2 in D (mean ± SD)	0.004 ± 0.109	0.02 ± 0.13	−0.04 ± 0.134
Astigmatism in D (mean ± SD)	0.006 ± 0.15	0 ± 0.098	−0.034 ± 0.18

Abbreviations: D, Dioptres; K1, corneal power in the flat meridian; K2, corneal power in the steep meridian; R1, radius of curvature in the flat meridian; R2, radius of curvature in the steep meridian.

### 3.2 K‐mean

**FIGURE 1:**
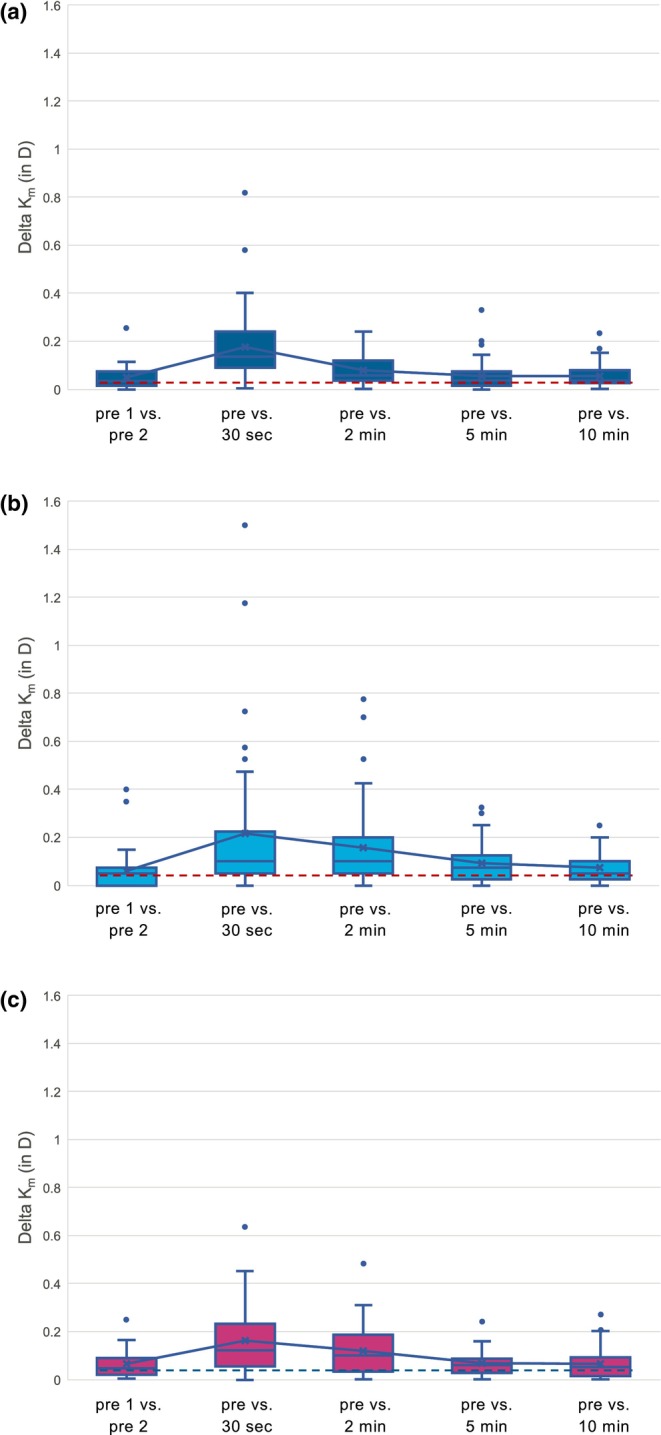
Comparative box plot analysis of the absolute differences in mean keratometry values (Delta K_m_) in dioptres (D) across different time intervals for three devices: The IOLMaster 700 (a), Pentacam AXL (b) and Galilei G6 (c). The *x*‐axis indicates the comparison pairs, starting with two preoperative measurements (Pre 1 vs. Pre 2), followed by post‐intervention (artificial tear application) intervals at 30 s, 2, 5 and 10 min. The *y*‐axis represents the absolute difference in k‐values in D.

The absolute changes in K_m_ after the application of ATs over time are shown in Figure 1 for each group. Detailed statistics are shown in Table 3. Measurements in Group I showed a significant difference in absolute K_m_ at the 30‐second (0.177 ± 0.021 D, *p* < 0.001) and two‐minute (0.079 ± 0.009 D, *p* = 0.004) marks compared to the baseline measurements (0.049 ± 0.008 D). Group P had a significant change in absolute K_m_ to the baseline measurement (0.061 ± 0.012 D) at 30 s (0.215 ± 0.041 D; *p* < 0.001), 2 min (0.157 ± 0.025 D, *p* < 0.001) and 5 min (0.094 ± 0.012 D, *p* = 0.007). Group G showed a significant difference in absolute K_m_ at 30 s (0.163 ± 0.02 D; *p* < 0.001) and 2 min (0.119 ± 0.014 D, *p* = 0.003) after AT application compared to the baseline measurements before the application (0.065 ± 0.009 D).

**TABLE 3:** Changes in K_m_ and astigmatism between measurements sorted by devices.

	*n*	|K_m_ (in D)|		Astigmatism (in D)	
		Mean ± SD	*p*	Mean ± SD	*p*
IOLMaster 700					
Pre 1 vs. Pre 2	48	0.049 ± 0.008		0.106 ± 0.015	
Pre vs. 30 sec		0.177 ± 0.021	<0.001*	0.200 ± 0.021	<0.001*
Pre vs. 2 min		0.079 ± 0.009	0.004*	0.152 ± 0.018	0.073
Pre vs. 5 min		0.056 ± 0.009	0.917	0.120 ± 0.017	0.505
Pre vs. 10 min		0.055 ± 0.007	0.325	0.089 ± 0.013	0.113
Pentacam AXL					
Pre 1 vs. Pre 2	49	0.061 ± 0.011		0.073 ± 0.009	
Pre vs. 30 sec		0.215 ± 0.041	<0.001*	0.220 ± 0.032	<0.001*
Pre vs. 2 min		0.157 ± 0.025	<0.001*	0.161 ± 0.028	0.001*
Pre vs. 5 min		0.094 ± 0.012	0.007*	0.092 ± 0.010	0.063
Pre vs. 10 min		0.074 ± 0.009	0.079	0.098 ± 0.014	0.093
Galilei G6					
Pre 1 vs. Pre 2	48	0.065 ± 0.009		0.125 ± 0.019	
Pre vs. 30 sec		0.163 ± 0.020	<0.001*	0.208 ± 0.025	0.004*
Pre vs. 2 min		0.119 ± 0.014	0.003*	0.144 ± 0.023	0.472
Pre vs. 5 min		0.069 ± 0.008	0.597	0.117 ± 0.013	0.926
Pre vs. 10 min		0.066 ± 0.009	0.939	0.115 ± 0.016	0.674

*Note*: The measurements Pre 1 and Pre 2 were taken before and the measurements 30 s, 2, 5 and 10 min taken after artificial tear application at the respective time intervals (*n* = 145); statistical significant values are marked with an asterisks.

Abbreviations: D, Dioptres; K_m_, mean corneal power; Pre 1, first measurement before artificial tear application; Pre 2, second measurement before artificial tear application.

When comparing the standard deviation of the different K_m_ measurements to the standard deviation between the two baseline measurements (−0.001 ± 0.073 D), statistical significance was shown at the 30‐second (−0.104 ± 0.208 D; *p* < 0.001) and 2‐min (0.012 ± 0.101 D; *p* = 0.013) marks of Group I. The standard deviation of the 5‐min (0.028 ± 0.079 D; *p* = 0.673) and 10‐min (0.008 ± 0.073 D; *p* = 0.523) measurements was not statistically significant compared to the baseline measurements. Similar findings were observed with Group G, which had a significantly different standard deviation of the 30‐s (0.02 ± 0.217 D; *p* < 0.001) and 2‐min (0.036 ± 0.152 D; *p* < 0.001) measurements compared to the standard deviation between the two baseline measurements (−0.026 ± 0.085 D). The K_m_ measurements of group P showed a statistically significant difference in standard deviation at the 30‐s (0.105 ± 0.341 D; *p* < 0.001), 2‐min (0.052 ± 0.228 D; *p* < 0.001) and 5‐min (−0.002 ± 0.125 D; *p* = 0.043) marks compared to the baseline measurements (0.016 ± 0.097 D). The ten‐minute measurement was not statistically significant (−0.04 ± 0.087 D; *p* = 0.713).

The three devices were tested against each other. Except for the 5‐min measurement between the IOLMaster 700 and the Pentacam AXL (*p* = 0.02), there were no significant differences between the devices regarding K_m_ (*p* > 0.05).

### 3.3 Astigmatism

At 30 s, 4.17% of eyes in Group I, 12.24% of eyes in Group P and 8.33% of eyes in Group G had a change in astigmatism >0.5 D. After 10 min of AT application, the number of eyes reduced to 0% in Group I and Group P, and 2.08% of eyes in Group G.

**FIGURE 2:**
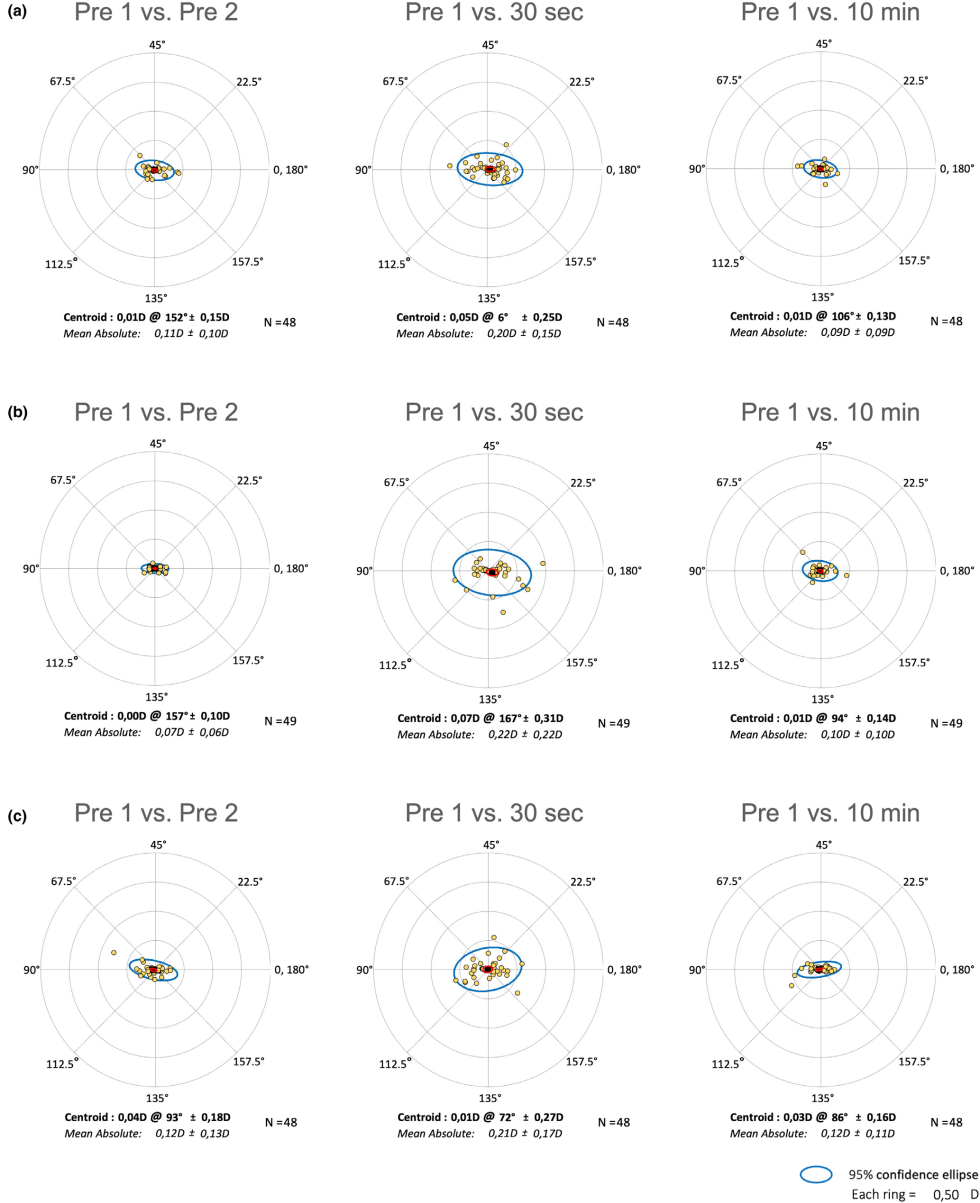
Vector analysis of astigmatism changes using double angle plots for three different devices: (a) IOLMaster 700, (b) Pentacam AXL and (c) Galilei G6. Each plot displays the centroid value of astigmatic change and the distribution of data points around it, for paired measurements taken at baseline (Pre 1 vs. Pre 2) and then at subsequent intervals (Pre 1 vs. 30 s, Pre 1 vs. 10 min). The centroid is presented in dioptres (D) with an axis and a standard deviation.

Group I showed a significant change in astigmatism between the measurements taken before AT application (0.106 ± 0.015 D) and the 30‐second measurement (0.2 ± 0.021 D, *p* < 0.001). Group P showed a difference in both the 30‐s measurement (0.22 ± 0.032, *p* < 0.001) and the 2‐min measurement (0.161 ± 0.028, *p* = 0.001) compared to the difference before AT application (0.073 ± 0.009). The difference in astigmatism in Group G was significantly different from the baseline measurement (0.125 ± 0.02) at the 30‐s mark (0.208 ± 0.025, *p* = 0.004). Figure 2 shows the change in astigmatism between the measurement without AT and 30 s and 10 min after AT application. Detailed statistics are shown in Table 3.

No significant difference was found between the three devices regarding astigmatism.

## 4 DISCUSSION

Our prospective clinical study, including 145 healthy patients, investigated the effects of AT on the corneal measurements of three different devices.

When observing the difference between the measurements before AT application, our study showed similar results to studies that primarily investigated the repeatability of these devices. For the IOLMaster 700, our study showed a mean difference in R1 of almost 0 mm and R2 of 0.001 mm, whereas Shajari et al. had a mean difference of 0.008 mm for R1 and 0.001 mm for R2 (Shajari et al., 2017). The mean difference in R1 and R2 in our Pentacam AXL group was −0.002 and −0.002 mm, respectively. Shajari et al. had a similar reliability with 0.005 and −0.003 mm (Shajari et al., 2017). In our study, the R1 and R2 measurements of the Galilei G6 (0.003 and 0.007 mm, respectively) were comparable with the results of Moshifar, who rounded to the second decimal place (−0.01 and −0.00 mm) (Moshirfar et al., 2022). This might indicate that a difference in corneal measurements after our intervention could be traced back to the AT application because this was the only parameter changed between measurements.

K_m_‐values were significantly different up to at least 2 min after AT application in each device. Group P showed a clinical significance for up to 5 min. The standard deviation was also significantly different between measurements at the same points in time, suggesting a higher variability. This might imply that corneal measurements shortly after AT application might not be as reliable. Except for a statistically significant difference in K values between the IOLMaster 700 and Pentacam AXL at the 5‐min measurements, there were no significant differences between the devices. A study by Fityo et al. observed Scheimpflug tomography to have a higher repeatability of astigmatism measurements compared to keratometry without any intervention (Fityo et al., 2016). This could not be observed in our study.

A few studies investigated the influence of AT on corneal parameters (Emde et al., 1996; Jensen et al., 2020; Nayer et al., 2021; Padmanabhan et al., 2022; Röggla et al., 2021). Compared to the abovementioned study by Röggla et al., our data show similar results (Röggla et al., 2021). While they used the IOLMaster 500, our study used the newer model IOLMaster 700 which uses a new method (SS‐OCT) as well as telecentric keratometry to measure corneal power. Our study used the measurement of the anterior surface using keratometry measurements. Röggla et al. observed a significant influence of eye drops on K‐readings, with both dry eyes and high viscosity AT causing a higher impact compared to normal eyes and low viscosity eye drops. We had similar results to their group with non‐dry eyes and low‐viscosity eye drops which closely resembles our patient cohort and the AT we used.

**FIGURE 3:**
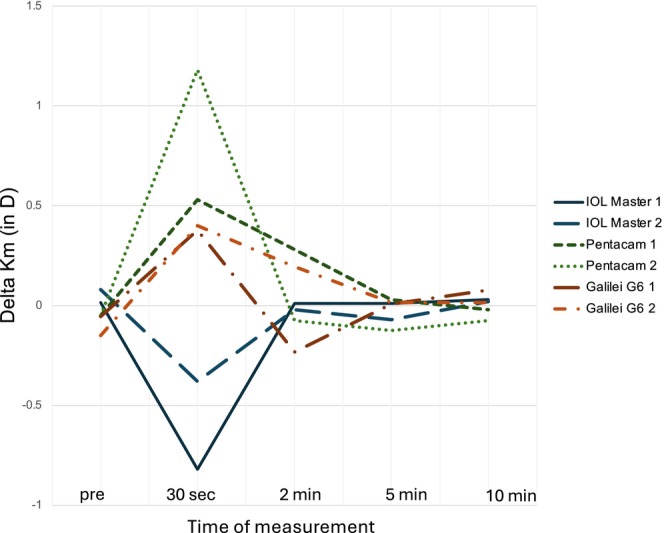
Line graph depicting outliers with significant mean delta keratometric power (Delta K_m_) in dioptres (D) from baseline across multiple time points: Initial measurement (pre) and subsequent measurements at 30 s, 2, 5 and 10 min post‐intervention (artificial tear application). The graph compares two iterations of measurements (1 and 2) for each device: IOLMaster 700, Pentacam AXL, and Galilei G6.

Every group had some patients with a high fluctuation in K_m_ (>1 D) after AT application over the different points in time (Figure 3). This might be especially relevant in cataract surgery because a difference of K_m_ of 1 D between measurements is roughly equivalent to a change of 1 D in IOL power (Zhang et al., 2020). Röggla et al. had a similar experience with outliers (Röggla et al., 2021). We could not identify a reason for these extreme changes in K_m_. One hypothesis might suggest the route causes an undiagnosed dry eye in these individuals, given that this has been shown to alter corneal parameters without the application of ATs (Epitropoulos et al., 2015).

A study by Jensen et al. looked at the influence of three different kinds of AT on the K‐value and its effects on IOL calculation in 38 patients undergoing cataract surgery (57 eyes) (Jensen et al., 2020). The measurements were taken prior to surgery and shortly after AT application with the IOLMaster 700. IOL calculation was performed using the Barrett TK Universal II formula and postoperative spherical equivalent (SEQ) was used as the outcome measure. Like us, they did not evaluate the tear film quality of the participants. They observed no significant influence of any AT type on K‐values and IOL power calculation. When looking at postoperative SEQ, the most precise results, although not significant, were achieved after saline eye drop application.

A different study by Emde et al. from 1996 investigated the influence of low‐ and high‐viscosity ATs on keratoscopy and 14 healthy eyes (Emde et al., 1996). They observed a significant alteration of astigmatism when using the high‐viscosity tear substitutes but not the low‐viscosity ones. This is similar to the findings of the study by Röggla et al. but different from ours (Röggla et al., 2021). Our use of low‐viscosity AT still produced significant alterations in K‐values while enrolling a higher number of patients. Comparison of these results should be done carefully as we did not use the same device (Emde et al., 1996).

Although the situations where lubricating ATs are applied shortly before an exam are sparse, there might be other scenarios where an eye drop is used prior to corneal measurements. Routine examination before cataract surgery can include biometry measurements of the cornea as well as a dilated fundus exam and measurements of intraocular pressure (IOP) (Espallargues et al., 1996). Applying the needed eye drops for these exams (mydriatic eye drops for pupil dilation or fluorescein eye drops for IOP measurements) before corneal measurements might also alter these results. A study by Padmanabhan et al. evaluated the influence of diagnostic drops like mydriatics and fluorescein on measurements taken by the Pentacam HR (Padmanabhan et al., 2022). While they observed no significant change in anterior or posterior corneal shape, they did detect a significant change in pachymetry and anterior chamber volume while using both kinds of drops. The second measurements were taken either 1.5 h or 10 min after intervention. From this, one could conclude that the influence might come from the pharmacological effects of these eye drops and not an acute change in tear film composition which might be the case in our patient cohort. A different study by Nayer et al. investigated the influence of multiple eye drops with diagnostic properties as well as Goldmann applanation tonometry on corneal measurements taken with a device using low coherence optical reflectometry (Lenstar LS900) (Nayer et al., 2021). They observed a significant change in central corneal thickness which they trace back to an increased tear film height. Anterior chamber depth and lens thickness were also significantly different. Nayer et al. theorize that this might be because of the mydriasis and cycloplegia and not the AT themselves. Despite these findings, the predicted post‐operative refraction was not affected by AT application.

The suggestion by Rochet et al. to use AT drops before corneal measurements to increase the quality needs to be further evaluated (Rochet et al., 2021). While the observations by Jensen et al. see an increase in reliability or at least no influence, our data and the study by Röggla et al. seem to indicate a lower quality (Jensen et al., 2020; Röggla et al., 2021).

Our study has some limitations. First, in contrast to Röggla et al., we only assessed potential dry eye with a brief patient history and not with objective testing (Röggla et al., 2021). Because of this, some patients might have tear film instability, which showed higher variance in some studies (Epitropoulos et al., 2015; Kundu et al., 2022). Secondly, we used only one type of AT substitute. Thus, extrapolation of this data to other kinds of eye lubricants might be limited. Especially because Röggla et al. and Emde et al. showed the highest influence on corneal measurements when using the high‐viscosity eye drops (Emde et al., 1996; Röggla et al., 2021).

Strengths of this study include the large number of included patients as well as the incorporation of three different devices that have different mechanisms of measurement. An additional strength is the observation period of 10 min after AT application. Röggla et al. showed an influence on K_m_ after the 5‐min measurement and therefore could not conclude a point in time when the cornea can be measured without a distortion of the values. We think this is especially relevant because Group P showed a significant difference in K_m_ after 5 min, but not after 10 min.

Further studies are needed with more patients, different kinds of ATs, and an objective testing of dry eye to confirm our results.

## 5 CONCLUSION

We recommend a waiting period of at least 5 min for the IOLMaster 700 and the Galilei G6 as well as a minimum of a 10‐min period for the Pentacam AXL after AT application for reduced variation in corneal parameter measurements.

## DISCLOUSE

Consultant, Research and Lecturing for Alcon, Schwind. Consultant and Lecturing for Oculus, Staar, Ziemer. Research and Lecturing for Teleon Surgical Consulting for Abbvie, Geuder, LensGen, Santen, Stadapharm, Thieme, Zeiss Meditec. Lecturing for Allergan, Bausch & Lomb, Johnson & Johnson, MedUpdate, streamedup, Teleon.

## CONFLICT OF INTEREST STATEMENT

T. Kohnen: Consultant, Research and Lecturing for Alcon, Schwind. Consultant and Lecturing for Oculus, Staar, Ziemer. Research and Lecturing for Teleon Surgical Consulting for Abbvie, Geuder, LensGen, Santen, Stadapharm, Thieme, Zeiss Meditec. Lecturing for Allergan, Bausch & Lomb, Johnson & Johnson, MedUpdate, streamedup, Teleon. All others have no conflict of interest to declare.

## ETHICS STATEMENT

This study includes human participants and was approved by the ethics committee of Goethe University Frankfurt (approval number: 20‐928). Before participating in the study, the participants gave their informed consent after being informed in detail.

## Data Availability

All data generated or analysed during this study are included in this article. Further enquiries can be directed to the corresponding author.
